# Natural Auxin Does Not Inhibit Brefeldin A Induced PIN1 and PIN2 Internalization in Root Cells

**DOI:** 10.3389/fpls.2019.00574

**Published:** 2019-05-09

**Authors:** Ivan A. Paponov, Tatyana Friz, Vadym Budnyk, William Teale, Florian Wüst, Martina Paponov, Salim Al-Babili, Klaus Palme

**Affiliations:** ^1^ Faculty of Biology, Institute of Biology II/Botany, Albert-Ludwigs-University of Freiburg, Freiburg, Germany; ^2^ Norwegian Institute of Bioeconomy Research (NIBIO), Division of Food Production and Society, Ås, Norway; ^3^ Faculty of Biology, Institute of Biology II/Cell Biology, Albert-Ludwigs-University of Freiburg, Freiburg, Germany; ^4^ Centre of Biological Systems Analysis, Albert-Ludwigs-University of Freiburg, Freiburg, Germany; ^5^ BIOSS Centre for Biological Signalling Studies, Albert-Ludwigs-University of Freiburg, Freiburg, Germany

**Keywords:** auxin, endocytosis, Brefeldin A (BFA), *Arabidopsis*, PIN-FORMED (PIN) proteins

## Abstract

The vesicle trafficking inhibitor Brefeldin A (BFA) changes the localization of plasma membrane localized PINs, proteins that function as polar auxin efflux carriers, by inducing their accumulation within cells. Pretreatment with the synthetic auxin 1-NAA reduces this BFA-induced PIN internalization, suggesting that auxinic compounds inhibit the endocytosis of PIN proteins. However, the most important natural auxin, IAA, did not substantially inhibit PIN internalization unless a supplementary antioxidant, butylated hydroxytoluene (BHT), was also included in the incubation medium. We asked whether the relatively small inhibition caused by IAA alone could be explained by its instability in the incubation solution or whether IAA might interact with BHT to inhibit endocytosis. Analysis of the IAA concentration in the incubation solution and of DR5 reporter activity in the roots showed that IAA is both stable and active in the medium. Therefore, IAA degradation was not able to explain the inability of IAA to inhibit endocytosis. Furthermore, when applied in the absence of auxin, BHT caused a strong increase in the rate of PIN1 internalization and a weaker increase in the rate of PIN2 internalization. These increases were unaffected by the simultaneous application of IAA, further indicating that endocytosis is not inhibited by the natural auxin IAA under physiologically relevant conditions. Endocytosis was inhibited at the same rate with 2-NAA, an inactive auxin analog, as was observed with 1-NAA and more strongly than with natural auxins, supporting the idea that this inhibition is not auxin specific.

## Introduction

Polar auxin transport is an essential process in the control of plant growth and development. Its regulation is mediated by the integration of auxin transporters into specific regions of the plasma membrane in a process that is widely understood to require the constant cycling of vesicles to and from the membrane (Geldner et al., 2001). This cycling requires endocytosis; although its existence was initially called into question in plants (Gradmann and Robinson, 1989), endocytosis is now well described throughout the eukaryotic domain (Doherty and McMahon, 2009). The rapid, actin-dependent cycling of PIN1 between plasma membrane and endosomal compartments was inferred after treatment of plants with the vesicle trafficking inhibitor Brefeldin A (BFA), which reversibly inhibited auxin efflux, spurring a resurgence of research into endocytosis in plant cells (Geldner et al., 2001; Kleine-Vehn and Friml, 2008). The localization of PIN proteins at the plasma membrane and in endomembrane compartments is well documented, with PIN proteins frequently being used as benchmarks for endocytosis and plasma membrane recycling. The distribution of PIN proteins between the plasma membrane and endomembrane compartments resulted in the demonstration that regulation of PIN endocytosis could be mediated by auxin itself (Paciorek et al., 2005). In this assay, pre-incubation of *Arabidopsis* seedlings with synthetic auxin analogs inhibited the formation of BFA compartments. The simultaneous application of different auxins and BFA led to the conclusion that the rate of internalization (endocytosis) of PIN proteins is negatively regulated by auxin itself. In this way, the amount of PIN proteins at the plasma membrane, and the rates of polar auxin transport, is increased (Paciorek et al., 2005). This model provided a mechanistic explanation for the feedback regulation of auxin transport by an auxin-mediated regulation of PIN abundance at the plasma membrane. Although subsequent characterization of the regulation of endocytosis by auxin gave indications for the involvement of auxin-binding protein 1 (ABP1) (Robert et al., 2010), recent evidence has indicated that ABP1 is neither involved in long-term (Gao et al., 2015) nor short-term auxin responses (Paponov et al., 2019). The molecular mechanisms of inhibition of endocytosis by auxin therefore remain poorly understood.

Using the photoconvertible fluorescent protein Dendra2 fused to PIN2, it was shown that, far from being static, BFA compartments are highly dynamic and contain not only internalized but also newly synthesized PIN proteins (Jasik et al., 2013). Auxin inhibits the accumulation of PIN2 in BFA compartments not by affecting rates of PIN2 internalization, but by suppressing the rate of PIN2 synthesis (Jasik et al., 2016). These data suggested that the regulation of PIN protein abundance at the plasma membrane might be different for different PIN proteins, suggesting an individual mechanistic analysis of PIN proteins might be important. Indeed, the abundance of different PIN proteins at the plasma membrane is not under the control of identical mechanisms. For example, the amount of PIN2 is regulated posttranscriptionally, with auxin stimulating PIN2 degradation *via* a mechanism not found for other PIN proteins (Abas et al., 2006).

Interestingly, in these studies, the effect of the synthetic auxin analog 1-NAA was always much stronger than the natural auxin IAA (Paciorek et al., 2005; Jasik et al., 2016). Somewhat surprisingly, in many of these studies the natural auxin IAA was not even used (Abas et al., 2006; Pan et al., 2009; Robert et al., 2010). In the study by Paciorek et al. (2005), the lack of an IAA effect at physiologically relevant concentrations levels was attributed to its instability in aqueous solution. However, and in contrast to the study of Paciorek et al. (2005), many other studies have been reported in which IAA remains active over periods of several days after its application to tissues at physiologically relevant concentrations (Eliasson et al., 1989; Woodward and Bartel, 2005; Rahman et al., 2007). This discrepancy raises the question as to whether, in the experiments reported by Paciorek et al. (2005), IAA was indeed instable or physiologically inactive. We therefore revisited these questions by re-analyzing the effects of auxins on BFA-induced PIN1 and PIN2 internalization and the stability of IAA in the incubation solution, finding that although 1-NAA does inhibit endocytosis, this property is not a general feature of auxinic compounds.

## Results

**Figure 1:**
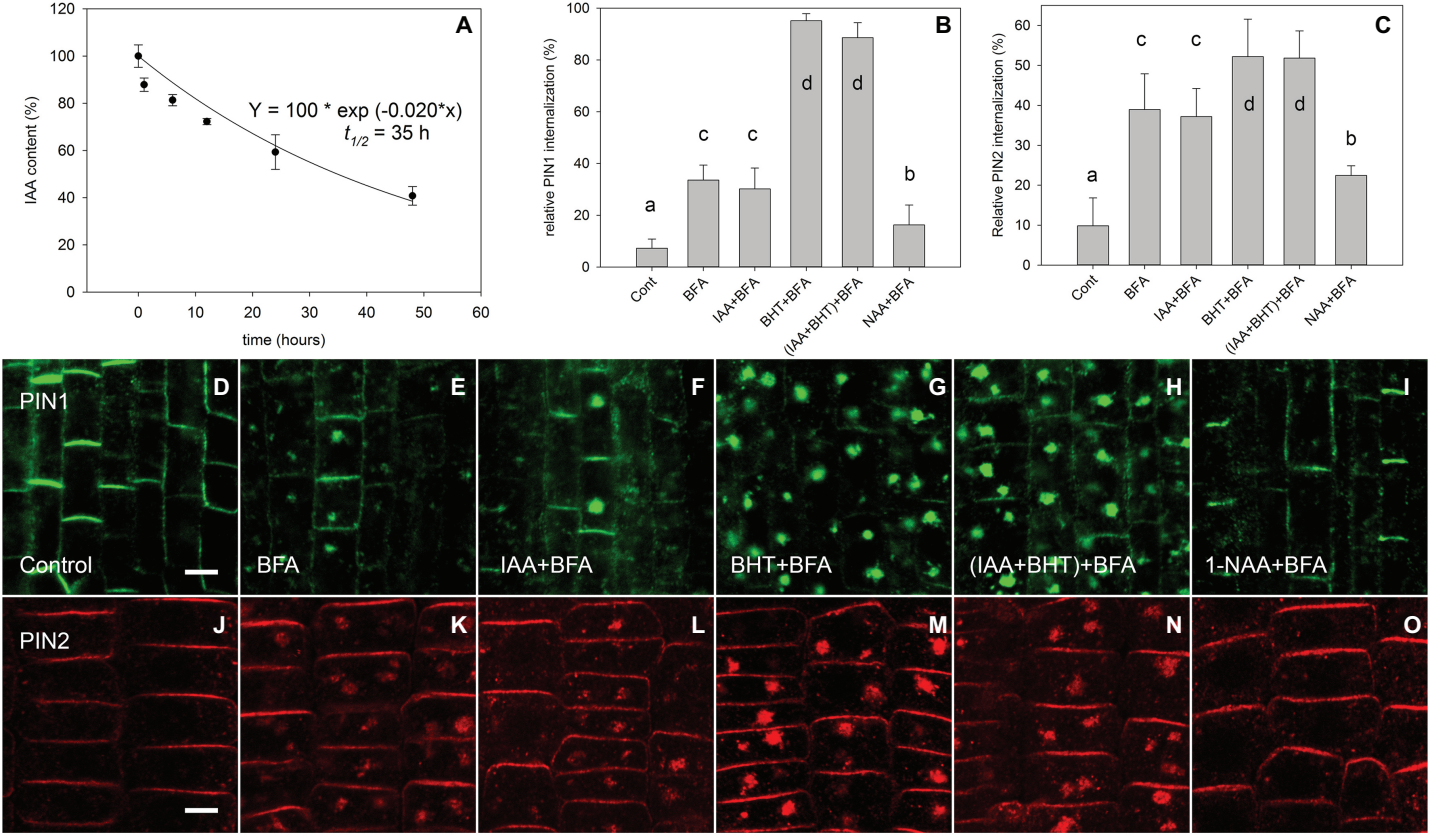
IAA stability in AM medium and the regulation of BFA-induced PIN1 and PIN2 internalization by auxins. **(A)** The effect of time on IAA concentration (% of starting amount) in the medium during incubation with *Arabidopsis* seedlings. Data shown are an average of three independent biological replicates; error bars represent SE. **(B,C)** Percentage of PIN1 and PIN2 internalization extracted by quantification of data represented in **(D–O)**. Quantitation of evaluation of PINs internalization was performed with Imaris 7.4 software (Bitplane AG) using surface reconstruction mode. Data are means of 4–9 seedlings; error bars represent SD. Means with different letters are significant (*p* < 0.05). Treatments were repeated in at least three independent experiments. **(D,J)** PIN1 and PIN2 localization under control conditions. **(E,K)** BFA (50 μM) induced PIN1 and PIN2 internalization. **(F,L)** IAA (10 μM) does not inhibit BFA-induced PIN1 and PIN2 internalization. **(G,M)** BHT stimulated BFA-induced PIN1 internalization and weakly stimulated PIN2 internalization. **(H,N)** simultaneous application of IAA and BHT has the same effect as BHT application for both PIN1 and PIN2. **(I,O)** 1-NAA (10 μM) inhibits BFA-induced PIN1 and PIN2 internalization. Scale bars represent 5 μm.

**Figure 2:**
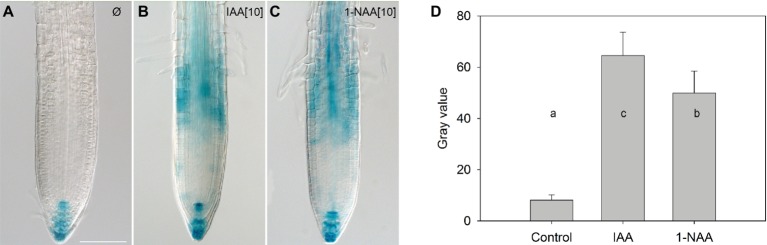
The effect of auxins on DR5-GUS expression in the roots of mock-treated 4-day-old seedlings. Seedlings were treated for 90 min with mock **(A)**, 10 μM IAA **(B)**, and 10 μM 1-NAA **(C)**, then transferred to a GUS staining solution and incubated for 220 min. Scale bars represent 100 μm. Quantification of GUS histochemical staining **(D)**. Data are means of nine seedlings; error bars represent SD. Means with different letters are significant (*p* < 0.05).

After immunolocalization of PIN1 and PIN2, we observed, in agreement with Paciorek et al. (2005), that at 10 μM, the synthetic auxin analog 1-NAA inhibited BFA-induced PIN internalization (Figures 1B–E,I–K,O). However, it did not share this property with the natural auxin IAA (Figures 1B–F,J–L). The absence of an IAA effect on endocytosis at 10 μM has previously been explained by an instability of IAA in the incubation medium (Paciorek et al., 2005). As this explanation is incompatible with well-documented IAA responses (Woodward and Bartel, 2005; Rahman et al., 2007), we directly analyzed IAA stability in the incubated medium by analyzing auxin content of culture media (Paciorek et al., 2005) by ultra-performance liquid chromatography (UPLC) followed by mass spectrometry (MS). Our data show that, under identical conditions to those reported by Paciorek et al. (2005), the half-life of IAA is 35 h (Figure 1A). This half-life is much longer than the reported degradation time of 30 min reported by Paciorek et al. (2005). We further confirmed the presence of active IAA throughout the experiment by measuring its ability to induce DR5-driven expression of beta-glucuronidase in roots (Figure 2; Ulmasov et al., 1997). We therefore conclude that IAA remained active throughout the experiment (which ran for 90 min), and that IAA degradation cannot explain its inability to inhibit endocytosis.

Interestingly, the simultaneous application of IAA and butylated hydroxytoluene (BHT) recovered IAA’s ability to inhibit BFA-induced PIN internalization (Paciorek et al., 2005), which was previously interpreted as the stabilization of IAA by BHT. However, as IAA is relatively stable in the assay medium (Figure 1A), the effect of BHT cannot be due to its stabilizing effect on IAA.

An alternative possibility is that BHT enhances the effect of auxin on endocytosis. We therefore estimated the effect of BHT alone and in combination with auxin on internalization of PIN1 and PIN2. We found that PIN proteins had a different sensitivity to BHT;BHT alone stimulated the internalization of PIN1, but had a weaker effect on the internalization of PIN2 (Figures 1B,C,G,H,M,N). Importantly, no interaction between BHT and auxin was found for PIN1 or for PIN2, supporting the hypothesis that BHT does not stabilize IAA or change its activity.

**Figure 3:**
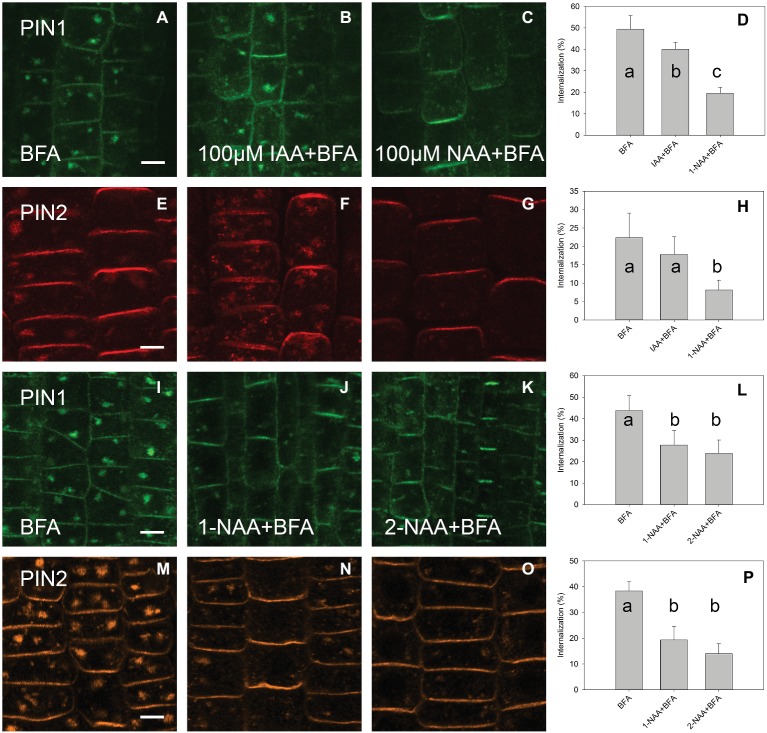
The effect of high auxin concentration and 2-NAA on BFA-induced PIN1 and PIN2 internalization. **(A,E,I,M)** PIN1 and PIN2 localization after BFA (50 μM) treatment. **(B)** IAA (100 μM) inhibited BFA-induced PIN1 internalization. **(F)** IAA (100 μM) did not significantly inhibit PIN2 internalization. **(C,G)** 1-NAA (100 μM) strongly inhibited PIN1 and PIN2 internalization. Data are means of 4–5 seedlings; **(D,H)** percentage of PIN1 and PIN2 internalization extracted by quantification of data represented in **(A–C)** and **(E–G)**, respectively. **(J–L)** 1-NAA and 2-NAA inhibit PIN1 internalization; **(N–P)** 1-NAA and 2-NAA inhibit PIN2 internalization. Scale bars represent 5 μm. Data are means of 7–9 seedlings; error bars represent SD. Means with different letters are significant (*p* < 0.05).

**Figure 4:**
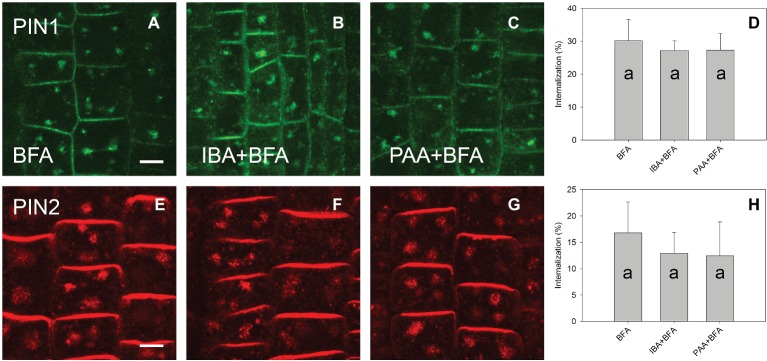
The effect of IBA and PAA on PIN1 and PIN2 internalization. **(A,E)** PIN1 and PIN2 internalization after BFA (50 μM) treatment. **(B–D)** IBA and PAA did not inhibit PIN1 internalization; **(F–H)** IBA and PAA did not inhibit PIN2 internalization. Scale bars represent 5 μm. Data are means of 8–9 seedlings; error bars represent SD. Means with different letters are significant (*p* < 0.05).

A high concentration of IAA (100 μM) reduced the level of PIN1 endocytosis; however, no significant reduction occurred for PIN2. At the same 100 μM concentration, 1-NAA strongly inhibited endocytosis (Figures 3A–H). Interestingly, 2-NAA, a physiologically inactive compound, inhibited PIN internalization to the same extent as was observed with 1-NAA (Figures 3I–P). At 10 μM, other auxins, including IBA and PAA, did not significantly reduce the rate of PIN internalization (Figure 4).

## Discussion

Most of the mechanistic models that describe auxin-dependent inhibition of endocytosis include ABP1 as a central factor (Pan et al., 2015). These models now require revision in light of the recent demonstration that ABP1 is neither involved in long-term (Gao et al., 2015) nor short-term auxin responses (Paponov et al., 2019). In this work, we provide evidence that the inhibition of endocytosis itself is independent of natural auxin at physiologically relevant concentrations. In our experiments, IAA did not inhibit endocytosis when applied at 10 μM, a concentration well above that required to inhibit root growth, indicating that the two processes are unlikely to be connected. Furthermore, the addition of antioxidant BHT to the medium increased PIN internalization, but did not change the activity of IAA.

Analysis of a number of functional analogs of IAA has shown that the inhibition of endocytosis and auxin-responsive gene regulation are regulated by independent mechanisms (Robert et al., 2010). For example, the auxin analog 5-fluoroindole-3-acetic acid (5-F-IAA) stimulates DR5-dependent gene expression but does not inhibit endocytosis (Robert et al., 2010). By contrast, another auxin analog, α-(phenyl ethyl-2-one)-indole-3-acetic acid (PEO-IAA), did not affect DR5-dependent reporter activity, but inhibited endocytosis (Robert et al., 2010). Such observations in which auxin-responsive gene expression and auxin-induced endocytosis can be decoupled support the hypothesis that different signaling pathways mediate both effects.

The fact that the most important natural auxin IAA has, even at high concentrations, a weak effect on endocytosis puts in doubt the categorization of auxin as a regulator of endocytosis. One possible explanation for the lack of IAA activity on endocytosis could be its instability in the nutrient solution (Paciorek et al., 2005). We are able to reject this hypothesis as IAA was stable in the nutrient solution in the course of our experiments (Figure 1A), and it maintained its activity throughout, as demonstrated by the activity of the DR5 auxin-responsive promoter (Figure 2). The observed stability of IAA in aqueous solution in our experiments is in general agreement with a large body of published data showing that the *in vitro* oxidative degradation of IAA takes days rather than minutes (Yamakawa et al., 1979; Dunlap et al., 1986; Dunlap and Robacker, 1988; Nissen and Sutter, 1990). Furthermore, IAA solutions retain their capacity to regulate root growth for as long as 48 h (Rahman et al., 2007).

The fact that 1-NAA inhibits endocytosis (Figures 1B,C,E,I,K,O; Paciorek et al., 2005) indicates that this synthetic auxin analog may have additional physiological properties that differ from those of natural auxins. This interpretation is supported by the fact that 2-NAA, an inactive auxin analog, showed the same activity in this regard as did 1-NAA. Importantly, the activity of 2-NAA was higher than that of any natural auxins tested in our experiment (Figure 4); thus, we cannot consider this activity to be auxin specific. The fact that 2-NAA has the same effect as 1-NAA precludes the possibility that the inhibition of endocytosis is stronger for the relatively lipophilic 1-NAA than for IAA as it more quickly penetrates into the cells. The inhibitory effect of 2-NAA on endocytosis does not agree with the original report (Paciorek et al., 2005), but it is supported by a more recent investigation that used different conditions for the BFA assay (specifically, 25 μM for both BFA and auxins) (Simon et al., 2013). Simon et al. (2013) found that 2-NAA inhibits endocytosis at the same rate as 1-NAA and more strongly than IAA; however, this observation did not raise concerns over the auxin specificity of this inhibitory effect.

Another argument used to support an inhibitory role for IAA is that endocytosis is suppressed in mutants that over-produce IAA (Paciorek et al., 2005). Specifically, the *superroot1 (sur1)* and *yucca* mutants showed a decreased PIN1 internalization after BFA treatment when compared to wild-type plants (Paciorek et al., 2005). However, the differences in PIN internalization among different auxin mutants might be the result of inherent structural differences caused by development under increased levels of endogenous auxin. Indeed, auxin signaling mutants have been used to show that rates of PIN internalization are changed by the effect of an altered sterol content in the plasma membrane (Pan et al., 2009). Another argument that auxin reduces PIN internalization is based on the observation that, after a change in the gravity vector, the BFA-induced internalization of PINs at the lower side of the root (where auxin accumulation is expected) was inhibited (Paciorek et al. 2005). Inhibiting the internalization of PIN2 might result in a higher abundance on the plasma membrane (Paciorek et al., 2005) and, respectively, a greater transport of auxin along the lower part of the root after a change in the gravity vector. However, less PIN2 has been observed on the lower side of the root, an observation attributed to the stimulation of PIN2 degradation by auxin (Abas et al., 2006). We should therefore be cautious when attributing gravity-dependent changes in the abundance of PIN2 on the plasma membrane to the exclusive effect of auxin on endocytosis, especially as it is not the only compound whose distribution is affected by gravity (Joo et al., 2001).

An investigation using the photoconvertible fluorescent protein Dendra2 to follow the movement of PIN2 highlights another feature of the regulation of PIN2 abundance by auxin, as here, auxin affected PIN2 biosynthesis more than it did the endocytosis pathway (Jasik et al., 2016). That said, a short-term effect (after 5 min) of auxin on endocytosis as observed by Robert et al. (2010) is difficult to explain exclusively by modulation of PIN2 synthesis.

The inhibition of endocytosis by synthetic molecules such as 1-NAA and 2-NAA raises questions about the mechanism of this regulation. As we are unable to hypothesize the presence of a dedicated NAA receptor, we find a direct modulation by NAA of the plasma membrane, the most parsimonious explanation. Indeed, an investigation with a plant (*A. thaliana*) membrane-mimicking system of manually mixed lipids showed that 1-NAA at a 10 μM concentration causes membrane destabilization, and this effect was stronger for 1-NAA than for IAA at the same concentration (Hac-Wydro and Flasinski, 2015).

Pharmacological research studies commonly report a phenomenon whereby synthetic compounds show a wider spectrum of activity than is observed for their natural counterparts (Feher and Schmidt, 2003). This clearly seems to be the case in the present study, as 1-NAA, at nonphysiological concentrations, elicits effects that seem to be unrelated to the action spectrum of IAA. Thus, any discovery of auxin-related processes based on the activity of a synthetic auxin should be carefully verified with a natural auxin at a physiological range of concentrations.

## Materials and Methods

*Arabidopsis thaliana* (L.) was grown on vertical orientated agar plates containing *Arabidopsis* medium (AM): half-strength MS salts and 1% sucrose, pH 5.7. Experiments were performed on 4-day-old seedlings in 24-well cell-culture plates in liquid AM medium. Seedlings were pre-treated for 30 min in AM with 10 μM IAA, 400 μg ml^−1^ BHT, 10 μM 1-NAA, 100 μM 1-NAA, 10 μM 2-NAA, 10 μM IBA, and 10 μM PAA. Pretreatments were followed by 45 min of concomitant treatment with auxins and 50 μM BFA. BFA was initially dissolved in dimethysulphoxide (DMSO) at 50 mM. Control treatments contained an equal amount of solvent.

Immunolocalization of *Arabidopsis* roots was analyzed as described (Friml et al., 2002). Rabbit anti-PIN1 (Gälweiler et al., 1998) and guinea pig anti-PIN2 (Tromas et al., 2009) were diluted at 1:500. The secondary antibodies, Alexa 488 and Alexa 555 conjugated anti-rabbit (for PIN1) and anti-guinea pig (for PIN2), were diluted at 1:400. Solutions during the immunolocalization procedures were changed using a pipetting robot (InsituPro; Intavis).

Confocal images were taken using a Zeiss LSM 510 NLO confocal laser scanning microscope. Alexa Fluor 488 was excited by applying a 488-nm argon laser line in combination with a 500–550 band-pass filter. Alexa Fluor 555 was excited by applying a helium-neon 543-nm laser (HeNe laser) in conjunction with a 575-long pass filter. Quantitative analysis of confocal microscopic images was performed using Imaris 7.5.6 software (Bitplane AG). The fluorescence signal was detected using the “create surface” tool, and the fluorescence signal at the plasma membrane and in the BFA bodies was calculated. The level of signal internalization (the signal in the BFA bodies) was calculated as the ratio between an intensity of intracellular fluorescence signal and the intensity of total fluorescence signal expressed as a percentage. For every root, the estimation of the level of PIN internalization was based on 20–32 and 10–18 cells for PIN1 and PIN2, respectively. We used 4–9 roots for every treatment. Averages for every root were used for the calculation of standard deviation. Student’s *t*-test was used for the evaluation of statistical significance between the experimental groups.

Four-day-old pDR5::GUS seedlings (Ulmasov et al., 1997) were pre-incubated in liquid AM medium supplemented or not with auxins (10 μM IAA or 10 μM 1-NAA) for 1.5 h and then transferred to the liquid medium and incubated for 1.5 h. They were subsequently stained for GUS activity in the following solution: 50 mM sodium phosphate buffer, pH 7.0, 5 mM potassium ferrocyanide, 5 mM potassium ferricyanide, 0.1% Triton X-100, and 1 mg/ml 5-bromo-4-chloro-3-indolyl glucuronide (X-Gluc) for 2.5 h at 37°C. Seedlings were then rinsed twice in phosphate buffer and subjected to an ethanol series: 20, 35, and 50% at the room temperature for 30 min. After stepwise rehydration in ethanol series, samples were mounted in a drop of clearing solution (chloral hydrate:water:glycerol, 8:3:1) (Weijers et al., 2001). Samples were viewed with a Zeiss AxioImager microscope equipped with differential interference contrast (DIC) optics using 20× dry objective. Quantification of GUS histochemical staining was done using ImageJ (Beziat et al., 2017).

UPLC analyses were performed using a Shimadzu UFLC-XR (Shimadzu, Japan) with a YMC Pack ProC18 column (150 mm × 3 mm) using a gradient program according to Kojima et al. (2009). The elution profile was traced by a fluorescence detector (Shimadzu RF-20A) with λex 280 nm and λem 350 nm. For identification, the corresponding peak was collected and used in MS analysis. Verification of the IAA peak was carried out by using a Finnigan-LTQ MS/MS (Thermo Scientific, Germany) with direct inject. IAA was detected in negative electron spay ionization mode (ESI) by monitoring the transition of the molecule ion 174.2 m/z to the main fragment 130 m/z.

## Author Contributions

IP and KP conceived the project. IP, KP, WT, and TF wrote the paper. TF, IP, VB, FW, MP, and SA-B performed the experiments and analyzed the data.

### Conflict of Interest Statement

The authors declare that the research was conducted in the absence of any commercial or financial relationships that could be construed as a potential conflict of interest.
